# Precision-cut rat, mouse, and human intestinal slices as novel models for the early-onset of intestinal fibrosis

**DOI:** 10.14814/phy2.12323

**Published:** 2015-04-23

**Authors:** Bao Tung Pham, Wouter Tobias van Haaften, Dorenda Oosterhuis, Judith Nieken, Inge Anne Maria de Graaf, Peter Olinga

**Affiliations:** 1 Department of Pharmacy, Pharmaceutical Technology and Biopharmacy, University of GroningenGroningen, The Netherlands; 2 Pathology Friesland FoundationLeeuwarden, The Netherlands; 3 Pharmacokinetics, Toxicology and Targeting, Department of Pharmacy, University of GroningenGroningen, The Netherlands

**Keywords:** Ex vivo model, intestinal fibrosis, precision-cut intestinal slice

## Abstract

Intestinal fibrosis (IF) is a major complication of inflammatory bowel disease. IF research is limited by the lack of relevant in vitro and in vivo models. We evaluated precision-cut intestinal slices (PCIS) prepared from human, rat, and mouse intestine as ex vivo models mimicking the early-onset of (human) IF. Precision-cut intestinal slices prepared from human (h), rat (r), and mouse (m) jejunum, were incubated up to 72 h, the viability of PCIS was assessed by ATP content and morphology, and the gene expression of several fibrosis markers was determined. The viability of rPCIS decreased after 24 h of incubation, whereas mPCIS and hPCIS were viable up to 72 h of culturing. Furthermore, during this period, gene expression of heat shock protein 47 and plasminogen activator inhibitor 1 increased in all PCIS in addition to augmented expression of synaptophysin in hPCIS, fibronectin (*Fn2*) and *TGF-β1* in rPCIS, and *Fn2* and connective tissue growth factor (*Ctgf*) in mPCIS. Addition of TGF-*β*1 to rPCIS or mPCIS induced the gene expression of the fibrosis markers *Pro-collagen1a1*, *Fn2,* and *Ctgf* in both species. However, none of the fibrosis markers was further elevated in hPCIS. We successfully developed a novel ex vivo model that can mimic the early-onset of fibrosis in the intestine using human, rat, and mouse PCIS. Furthermore, in rat and mouse PCIS, TGF-*β*1 was able to even further increase the gene expression of fibrosis markers. This indicates that PCIS can be used as a model for the early-onset of IF.

## Introduction

Intestinal fibrosis (IF) is a major complication that can occur in inflammatory bowel disease (IBD), after radiation therapy or transplantation. IF is a result of chronic inflammation or injury and originates from inflammatory and immune processes acting simultaneously on many different cell types (Rieder and Fiocchi 2009). The imbalance between inflammation/injury and tissue repair leads to excessive accumulation of collagen, fibronectin, and other extracellular matrix (ECM) proteins (Rieder et al. 2007). Due to the luminal structure of the intestine, thickening of its wall by fibrosis causes stenosis, eventually requiring surgical intervention (Froehlich et al. 2005). In Crohn's Disease (CD), progressive IF leads to symptomatic bowel strictures and stenosis due to narrowing of the lumen in 30% of patients (Silverstein et al. 1999). Despite major pharmacological advances in the inflammatory component of the disease, the incidence of stricture formation in CD has not markedly changed in the past 10 years (Latella et al. 2013). CD patients that barely suffer from inflammation can still have extensive degrees of fibrosis and stenosis and vice versa (Louis et al. 2001). These findings suggest that distinct mechanisms of inflammation and restitution/fibrosis exist. Up to now, the mechanism underlying IF is still not fully understood and there is no pharmacological therapy to prevent and/or cure the fibrotic state.

Lack of knowledge about the mechanism of IF is a considerable limitation in developing antifibrotic drugs because suitable drug targets need to be unraveled. Furthermore, the currently used (human) in vitro and animal in vivo models, mainly rodent, are not representative for the (patho)physiology of IF in human (Fiocchi and Lund 2011). The in vivo animal models provoke substantial discomfort to the animals and require large numbers of animal experiments (Rieder et al. 2012). In addition, available in vitro and cell-culture models cannot imitate the physiologic milieu, especially not cell–cell and cell–extracellular matrix interactions between fibroblasts, stellate cells, bone-marrow-derived cells, fibrocytes, and pericytes (Rieder and Fiocchi 2008). One of the most important profibrotic cytokines in IF is transforming growth factor-*β*1 (TGF-*β*1) (Lund and Rigby 2008). Through the phosphorylation of Smad proteins, TGF-*β*1 can activate the downstream signaling including the expression of plasminogen activator inhibitor 1 (PAI-1) (Kutz et al. 2001). Many of its downstream effects leading to deposition of extracellular matrix (ECM), are mediated by connective tissue growth factor (CTGF) (Dendooven et al. 2011; Latella et al. 2013). Phenotypically altered resident fibroblasts can turn into myofibroblasts that start to express alpha-smooth muscle actin (*α*SMA) and to produce excessive amounts of ECM. This ECM mainly consists of collagen, fibronectin (FN2), and elastin (ELA) (To and Midwood 2011). The maturation of collagen is facilitated by heat shock protein 47 (HSP47*)* for which collagen is the only substrate (Taguchi and Razzaque 2007). Furthermore, stellate cells which have been confirmed to play a role in liver fibrosis (Synaptophysin (SYN) is a marker), are proposed to play a role in intestinal fibrosis (Cassiman et al. 1999; Van de Bovenkamp et al. 2006; Fiocchi and Lund 2011). To be able to study the complex interplay between various intestinal cell types, an ex vivo (human) system which can mimic the multicellular process, namely precision-cut intestinal slices (PCIS), was used (De Graaf et al. 2010). PCIS have been used as a model to study drug metabolism (Van de Kerkhof et al. 2008), xenobiotic interactions, and drug transport (Vickers and Fisher 2005; Possidente et al. 2011; Niu et al. 2013). Furthermore, precision-cut tissue slices from various organs have been successfully used as a model to study fibrosis and the efficacy of antifibrotic compounds (Westra et al. 2013). In PCIS, all intestinal cell types are kept in their original tissue-matrix environment and structure. Thus, cell–cell and cell-ECM interactions are retained. Furthermore, the villus and microvillus organization is preserved, which is essential for the migration and transformation of intestinal cells (de Graaf et al. 2007; Niu et al. 2013; Westra et al. 2013).

The aim of this study was to evaluate PCIS prepared from human, rat, and mouse, as a novel model to mimic the early-onset of (human) IF. This model could be used to unravel the mechanism of intestinal fibrosis as well as to test the efficacy of antifibrotic compounds ex vivo. First, the viability and morphology of PCIS were studied during culture. Second, the gene expressions of above mentioned fibrosis markers (*CTGF, αSMA, Pro-collagen 1a1 (COL1A1), FN2, HSP47, ELA, PAI-1, TGF-β1,* and *SYN*) were determined in PCIS in the presence and absence of the fibrogenic factor TGF-*β*1.

## Material and Methods

### Preparation of rat and mouse intestinal cores

Adult nonfasted male Wistar rats and C57BL/6 mice were used (Harlan PBC, Zeist, The Netherlands). The rats and mice were housed on a 12 h light/dark cycle in a temperature and humidity controlled room with food (Harlan chow no 2018, Horst, The Netherlands) and water ad libitum. The animals were allowed to acclimatize for at least seven days before the start of the experiment. The experiments were approved by the Animal Ethical Committee of the University of Groningen.

Rats and mice were anesthetized with isoflurane/O_2_ (Nicholas Piramal, London, UK). Rat jejunum (about 25 cm distal from the stomach and 15 cm in length) and mouse jejunum (about 15 cm distal from the stomach and 10 cm in length) were excised and preserved in ice-cold Krebs-Henseleit buffer (KHB) supplemented with 25 mm d-glucose (Merck, Darmstadt, Germany), 25 mm NaHCO_3_ (Merck), 10 mm HEPES (MP Biomedicals, Aurora, OH), saturated with carbogen (95% O_2_/5% CO_2_) and adjusted to pH 7.4. The jejunum was cleaned by flushing KHB through the lumen and subsequently divided into 2 cm segments. These segments were filled with 3% (w/v) agarose solution in 0.9% NaCl at 37°C and embedded in an agarose core-embedding unit (De Graaf et al. 2010).

### Preparation of human intestinal cores

Healthy human jejunum tissue was obtained for research from intestine that was resected from patients who underwent a pancreaticoduodenectomy (Table1). The experimental protocols were approved by the Medical Ethical Committee of the University Medical Center Groningen.

**Table 1:** Characteristics of human PCIS from nine Human donors.

Human ID	Gender	Age	ATP (0 h) pmol/*μ*g protein
IH1	F	73	4.70
IH2	F	80	1.60
IH3	M	68	1.70
IH4	F	66	7.90
IH5	M	33	2.30
IH6	F	66	4.18
IH7	M	53	5.36
IH8	F	71	3.10
IH9	M	53	3.71

The healthy jejunum was preserved in ice-cold KHB until the embedding procedure (De Graaf et al. 2010; Roskott et al. 2010). The submucosa, muscularis, and serosa were carefully removed from the mucosa within an hour after collection of the tissue. The mucosa was divided into 0.4 cm × 1 cm sheets. These sheets were embedded in 3% agarose (w/v) solution in 0.9% NaCl at 37°C and inserted in embedding unit (De Graaf et al. 2010).

### Preparation of PCIS

PCIS were prepared in ice-cold KHB by the Krumdieck tissue slicer (Alabama Research and Development). The slices with a wet weight of 3–4 mg had an estimated thickness of 300–400 *μ*m (De Graaf et al. 2010). Slices were stored in ice-cold KHB until the start of the experiments (De Graaf et al. 2010).

### Incubation of intestinal slices

Slices were incubated in 12-well plates for human PCIS (hPCIS) and rat PCIS (rPCIS) or in 24-well plates for mouse PCIS (mPCIS). hPCIS and rPCIS were incubated individually in 1.3 mL and mPCIS in 0.5 mL of Williams Medium E with L-glutamine (Invitrogen, Paisly, UK) supplemented with 25 mm glucose, 50 *μ*g/mL gentamycin (Invitrogen), and 2.5 *μ*g/mL amphotericin-B (Invitrogen). During incubation (at 37°C and 80% O_2_/5% CO_2_) in an incubator (MCO-18M, Sanyo), the plates were horizontally shaken at 90 rpm (amplitude 2 cm). rPCIS were incubated up to 24 h, mPCIS and hPCIS were incubated up to 72 h, with and without human TGF-*β*1 (Roche Diagnostics, Mannheim, Germany) in the concentration range from 1 to 10 ng/mL. All incubations were performed manifold (using 3–6 slices incubated individually in separate wells) and were repeated with intestine from 3 to 16 different humans, rats, or mice.

### Viability and morphology

The viability was assessed by measuring the adenosine triphosphate (ATP) content of the PCIS, as was previously described (De Graaf et al. 2010). Briefly, after incubation, slices were transferred to 1 mL sonication solution (containing 70% ethanol and 2 mm EDTA), snap-frozen in liquid nitrogen and stored at −80°C. To determine the viability, ATP levels were measured in the supernatant of samples sonicated for 45 sec and centrifuged for 2 min at 4°C at 16.000 × g, using the ATP bioluminescence kit (Roche Diagnostics, Mannheim, Germany). ATP values (pmol) were normalized to the total protein content (*μ*g) of the PCIS estimated by Lowry method (BIO-rad RC DC Protein Assay, Bio Rad, Veenendaal, The Netherlands). Values displayed are relative values compared to the related controls.

To assess the morphology, incubated slices were fixed in 4% formalin and embedded in paraffin. Sections of 4 *μ*m were cut and stained with hematoxylin and eosin (HE) (De Graaf et al. 2010). HE sections were scored according to a modified Park score, describing the sequence of development of tissue injury in the intestine after ischemia and reperfusion (Park et al. 1990; Roskott et al. 2010). The integrity of seven segments of the PCIS was scored on a scale from 0 to 3. Viability of the epithelium, stroma, crypts, and muscle layer were scored separately rating 0 if there was no necrosis, and 3 if massive necrosis was present. The other parts of the intestinal slice were rated as follows: Shape of the epithelium: 0 = cubic epithelium, 3 = more than 2/3 of the cells are flat, flattening of the villi: 0 = normal, 3 = more than 2/3 of the villi are flattened, and the amount of edema: 0 = no edema, 3 = severe edema. A maximum score of 21 indicates severe damage. In human samples, the morphological score of muscularis mucosae was determined in the “muscle layer” section. B.T.P., W.T.v.H. and J.N. performed the blind scoring; the mean of three total scores was calculated.

### Gene expression

After incubation, slices were snap-frozen in liquid nitrogen and stored at −80°C until RNA isolation. First, total RNA of three to six pooled snap-frozen slices was isolated using Qiagen RNAeasy mini kit (Qiagen, Venlo, The Netherlands). The amount of isolated RNA was measured with the BioTek Synergy HT (BioTek Instruments, Vermont). Afterward, reverse transcriptase was performed with 1 *μ*g RNA using Reverse Transcription System (Promega, Leiden, The Netherlands). The reverse transcript polymerase chain reaction (PCR) reaction was performed in the Eppendorf mastercycler with the following gradient: 25°C for 10 min, 45°C for 60 min, and 95°C for 5 min.

The expression of the fibrosis genes, namely *COL1A1*, *αSMA*, *HSP47*, *CTGF*, *FN2, TGF-β1, PAI-1,* and *SYN* were determined by either the Taqman or SYBRgreen method. In hPCIS, ELA gene expression was also measured by SYBRgreen method. With the Taqman method, the primers (50 *μ*m) and probes (5 *μ*m) listed in Table2 were used with the qPCR mastermix plus (Eurogentec, Maastricht, The Netherlands). The real-time PCR reaction was performed in a 7900HT Real Time PCR (Applied Biosystems, Bleiswijk, The Netherlands) with 40 cycles of 10 min at 95°C, 15 sec at 95°C and 1 min at 60°C. With the SYBRgreen method, appropriate primers (50 *μ*m), listed in Table2, were used with SYBRgreen mastermix (GC Biotech, Alphen aan de Rijn, The Netherlands). The real-time PCR reaction was performed with 45 cycles of 10 min 95°C, 15 sec at 95°C, and 25 sec at 60°C following with a dissociation stage. Ct values were corrected for the Ct values of the housekeeping gene *GAPDH* (ΔCt) and compared with the control (ΔΔCt). Results are calculated as fold induction of the gene (2^−ΔΔCt^).

**Table 2:** Primers and probes of fibrosis markers.

Primers/Probe	Forward	Reverse	Probe
Mouse			
* Gapdh*	ACAGTCCATGCCATCACTGC	GATCCACGACGGACACATTG	
* Col1a1*	TGACTGGAAGAGCGGAGAGT	ATCCATCGGTCATGCTCTCT	
* αSma*	ACTACTGCCGAGCGTGAGAT	CCAATGAAAGATGGCTGGAA	
* Hsp47*	AGGTCACCAAGGATGTGGAG	CAGCTTCTCCTTCTCGTCGT	
* Ctgf*	CAAAGCAGCTGCAAATACCA	GGCCAAATGTGTCTTCCAGT	
* Fn2*	CGGAGAGAGTGCCCCTACTA	CGATATTGGTGAATCGCAGA	
* Syn*	CTGTGTTTGCCTTCCTCTACTC	AGGTAGGGCTCAGACAGATAAA	
* Tgf-β1*	GGTTCATGTCATGGATGGTGC	TGACGTCACTGGAGTTGTACGG	
* Pai-1*	GCCAGATTTATCATCAATGACTGGG	GGAGAGGTGCACATCTTTCTCAAAG	
Rat			
* Gapdh*	GAACATCATCCCTGCATCCA	CCAGTGAGCTTCCCGTTCA	CTTGCCCACAGCCTTGGCAGC
* Col1a1*	CCCACCGGCCCTACTG	GACCAGCTTCACCCTTAGCA	CCTCCTGGCTTCCCTG
* αSma*	AGCTCTGGTGTGTGACAATGG	GGAGCATCATCACCAGCAAAG	CCGCCTTACAGAGCC
* Hsp47*	AGACGAGTTGTAGAGTCCAAGAGT	ACCCATGTGTCTCAGGAACCT	CTTCCCGCCATGCCAC
* Ctgf*	ACACAAGGGTCTTCTGCGA	TTGCAACTGCTTTGGAAGGAC	
* Fn2*	TCTTCTGATGTCACCGCCAACTCA	TGATAGAATTCCTTGAGGGCGGCA	
* Syn*	CTTTGCCATCTTCGCCTTTG	GCCCGTAATCGGGTTGATAA	
* Tgf-β1*	CCTGGAAAGGGCTCAACAC	CAGTTCTTCTCTGTGGAGCTGA	
* Pai-1*	AACCCAGGCCGACTTCA	CATGCGGGCTGAGACTAGAAT	
Human			
* GAPDH*	ACCAGGGCTGCTTTTAACTCT	GGTGCCATGGAATTTGCC	TGCCATCAATGACCCCTTCA
* COL1A1*	CAATCACCTGCGTACAGAACGCC	CGGCAGGGCTCGGGTTTC	CAGGTACCATGACCGAGACGTG
* αSMA*	AGGGGGTGATGGTGGGAA	ATGATGCCATGTTCTATCGG	GGGTGACGAAGCACAGAGCA
* HSP47*	GCCCACCGTGGTGCCGCA	GCCAGGGCCGCCTCCAGGAG	CTCCCTCCTGCTTCTCAGCG
* FN2*	AGGCTTGAACCAACCTACGGATGA	GCCTAAGCACTGGCACAACAGTTT	GGGCACCACCAAGGTC
* CTGF*	ACGGCGAGGTCATGAAGAAGAACA	ACTCTCTGGCTTCATGCCATGTCT	
* SYN*	CTGCACCAAGTGTACTTTGAT	GCTGACGAGGAGTAGTCCC	
* TGF-β1*	TGGCGATACCTCAGCAACC	CTCGTGGATCCACTTCCAG	
* PAI-1*	CACGAGTCTTTCAGACCAAG	AGGCAAATGTCTTCTCTTCC	
* ELA*	GGCCATTCCTGGTGGAGTTCC	AACTGGCTTAAGAGGTTTGCCTCCA	

### Statistics

A minimum of three different intestines was used for each experiment, using 3–6 slices from each intestine. The results are expressed as mean ± standard error of the mean (SEM). Differences were determined using a paired, one-tailed Student's *t*-test and ANOVA multiple comparisons with Fisher's least significant difference test. A *P*-value <0.05 was considered significant. Statistical differences in ATP were determined using the values relative to the control values in the same experiment. Real-time PCR results were compared using the mean ΔΔCt values. Correlation between ATP content and mean Park score was determined using Spearman's correlation coefficient.

## Results

### Viability of PCIS

**Figure 1:**
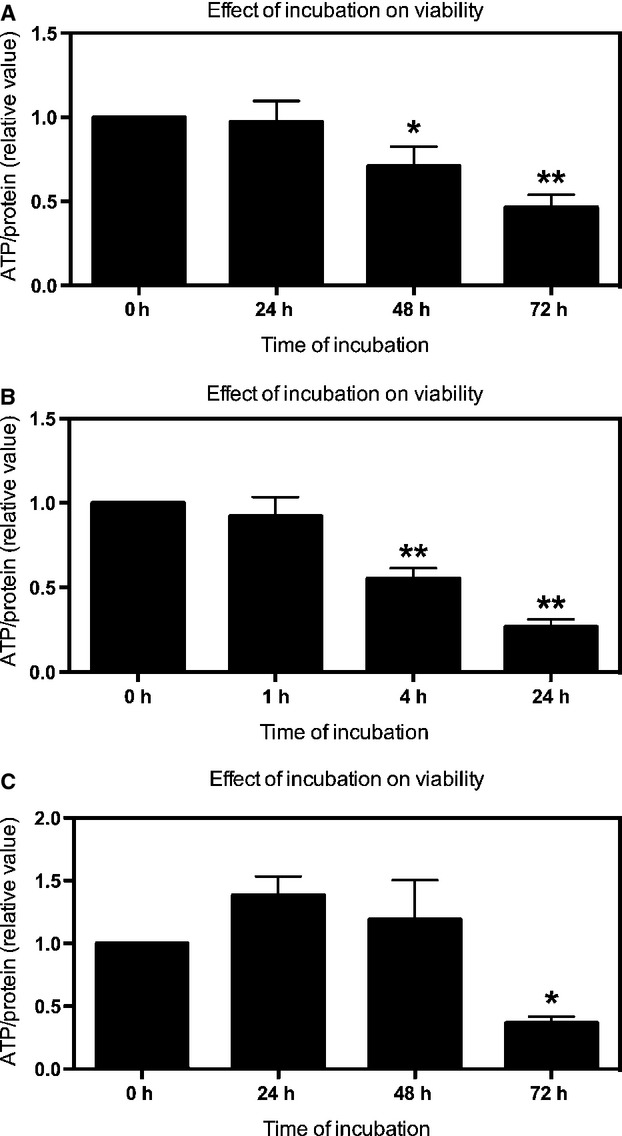
Long-term incubation of hPCIS, rPCIS, and mPCIS. The viability of PCIS as measured by ATP content (relative value compare to 0 h) after incubation of (A) hPCIS up to 72 h, (B) rPCIS up to 24 h, and (C) mPCIS up to 72 h. (*P <* *0.05, **P <* *0.01 vs. 0 h. *n* = 9–15, data are expressed as mean ± SEM).

The ATP content and the morphology of PCIS were used to evaluate the viability of the slices during culturing. Directly after slicing, the ATP content of the PCIS from human (h), rat (r) and mouse (m) was 3.49 ± 1.56, 4.34 ± 1.69, 3.82 ± 1.95 pmol/*μ*g protein, respectively. No significant difference in the ATP content of PCIS from different species was found. When compared with directly after slicing, the ATP content of hPCIS decreased about 30% and 50%, after 48 and 72 h of incubation, respectively (Fig.1A). However, in rPCIS, already after 4 h of incubation the ATP content significantly decreased and after 24 h the ATP content was reduced by 75% compared to freshly prepared PCIS (Fig.1B). In mPCIS, the ATP content was not significantly different after 48 h of incubation, yet, after 72 h of culturing, ATP levels were significantly decreased to 36% as compared to freshly prepared PCIS (Fig.1C).

**Figure 2:**
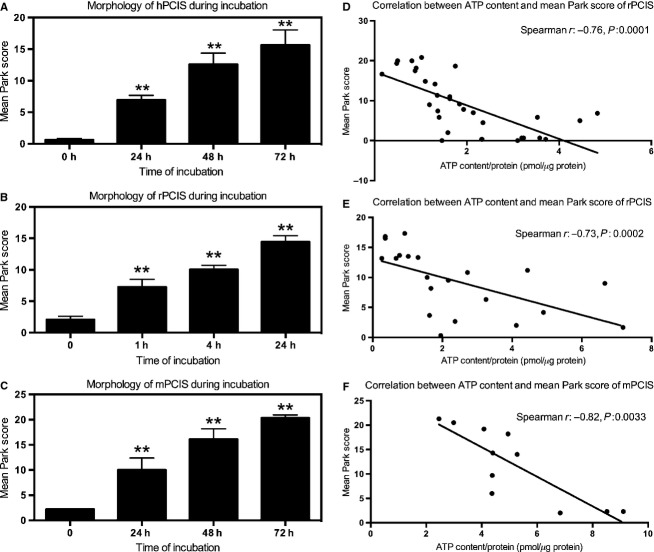
Park score of hPCIS, rPCIS, and mPCIS after long-term incubation. Mean Park scores of (A) hPCIS, (B) rPCIS, and (C) mPCIS after different incubation intervals. (by BTP, JN, and WTvH, *P <* *0.05, *n* = 5–8, data are expressed as mean +/- SEM). (D) Spearman correlation between ATP content and mean Park score in hPCIS (*r* = −0.76, *P *<* *0.0001), (E) rPCIS (*r* = −0.73, *P *≤ 0.0001) and (F) mPCIS (*r* = −0.81, *P *=* *0.0015).

To evaluate the morphological integrity of PCIS after incubation, the modified Park score was used. An increased Park score indicated a decrease in viability. Mean Park scores of hPCIS increased significantly during 48 h of culture when compared with hPCIS directly after slicing (Fig.2A). Furthermore, the mean Park score of rPCIS and mPCIS also increased significantly during incubation (Fig.2B and C). Very low Park score of nonincubated slices showed that PCIS were not damaged by handling and slicing (Fig.2). A significant correlation between ATP content and mean Park scores was found in hPCIS (Spearman *r* = −0.76, *P *<* *0.0001), rPCIS (Spearman *r* = −0.73, *P *≤ 0.0002) and mPCIS (Spearman *r* = −0.82, *P *=* *0.0033) (Fig.2D–F).

**Figure 3:**
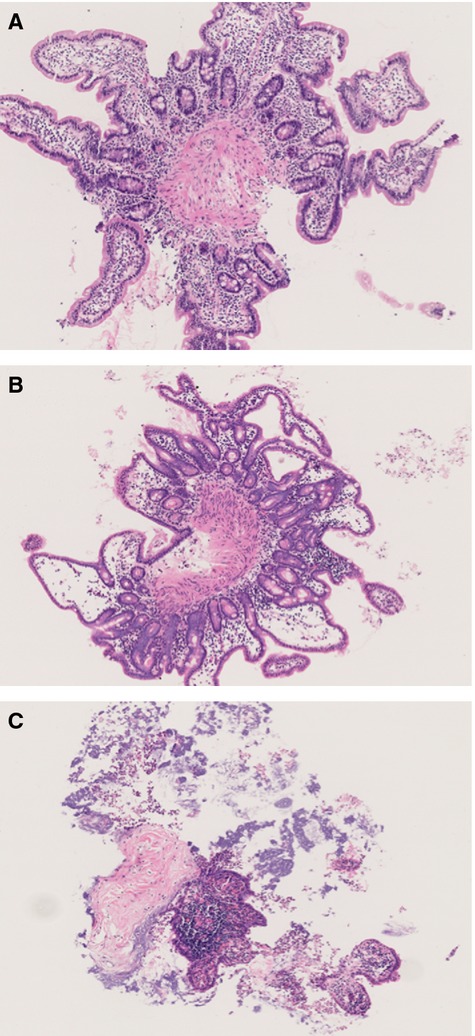
Morphological integrity of hPCIS after long-term incubation. HE staining of representative healthy hPCIS after (A) 0 h, (B) 48 h, and (C) 72 h incubation (magnification: 4×).

During culturing of human, rat, and mouse intestinal slices the same sequence of morphological changes and damage was found (Fig.3). First, epithelial and stromal cells were damaged, with clear signs of necrosis in these cells. In association, flattening of the villi and of epithelial cells (i.e., losing their cubic shape), and development of edema was found (Fig.3B). Subsequently, necrosis was evident in cells of the crypts and the muscle layer. By incubating hPCIS up to 72 h, massive necrosis was apparent in both epithelial and stromal cells (Fig.3C). In association with necrosis, destruction of the normal tissue architecture was found as shown in Figure3C.

**Figure 4:**
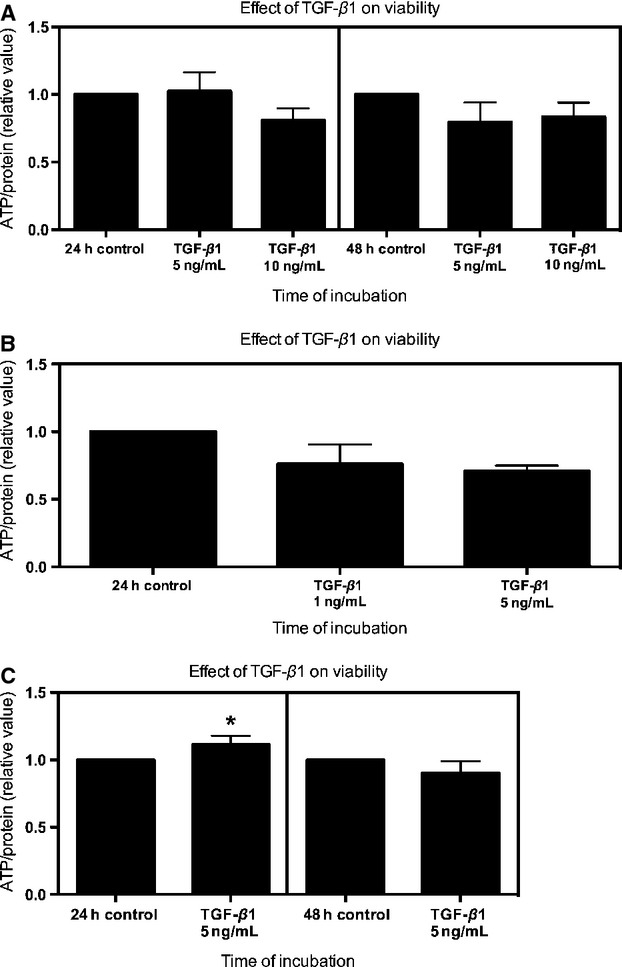
Long-term incubation of hPCIS, rPCIS, and mPCIS with TGF-*β*1. The viability of PCIS as measured by ATP content (relative value compare to control) after incubation of: (A) hPCIS up to 48 h with 5 ng/mL and 10 ng/mL TGF-*β*1, (B) rPCIS up to 24 h with 1 ng/mL and 5 ng/mL TGF-*β*1 and (C) mPCIS up to 48 h with 5 ng/mL TGF-*β*1. (*P <* *0.05, **P <* *0.01 vs. control *n* = 3–6, data are expressed as mean ± SEM).

PCIS of all species were incubated with TGF-*β*1, to confirm that PCIS can be used to study the TGF-*β*1 signaling pathway. hPCIS viability decreased slightly, but not significantly, after 24 h of incubation with 10 ng/mL TGF-*β*1. Meanwhile, when hPCIS were incubated for 48 h, TGF-*β*1 did not affect the hPCIS ATP content (Fig.4A). Moreover, ATP content of rPCIS did not decreased after 24 h of incubation in the presence of up to 5 ng/mL TGF-*β*1 (Fig.4B). In contrast, 10 ng/mL of TGF-*β*1 decreased the viability of rPCIS considerably (data not shown). Meanwhile, in mPCIS, up to 48 h in culture, no effect on the viability due to TGF-*β*1 was observed (Fig.4C).

### Gene expression of fibrosis markers

**Figure 5:**
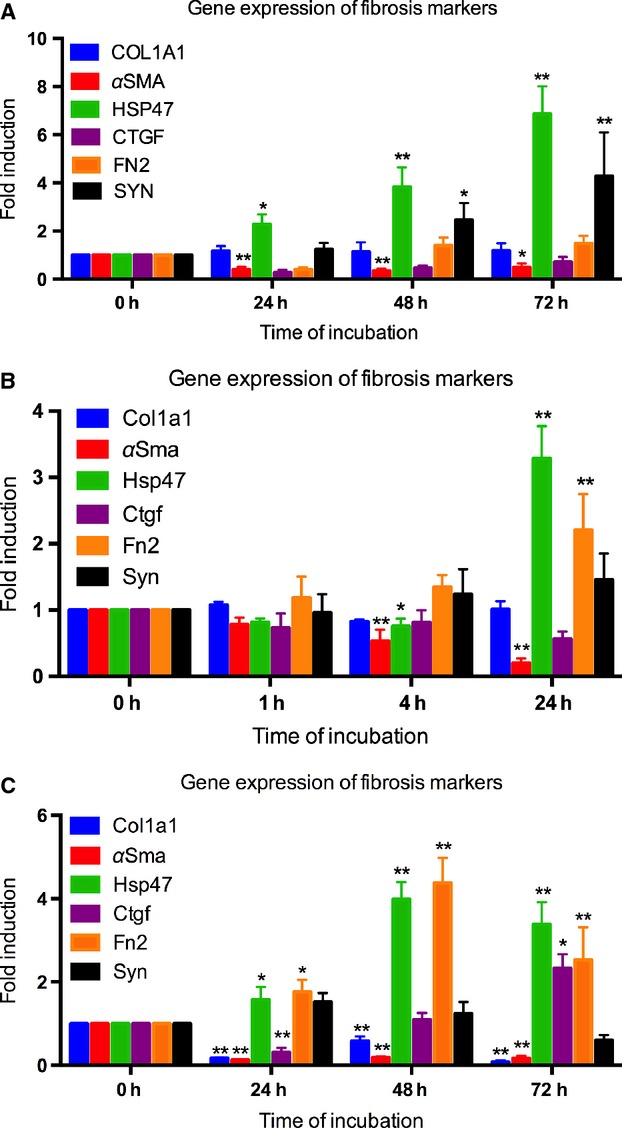
Gene expression of fibrosis markers in hPCIS, rPCIS, and mPCIS after long-term incubation. The gene expression of fibrosis markers *COL1A1*, *HSP47*, *αSMA*, *CTGF*, *FN2,* and *SYN* after incubation of (A) hPCIS for 72 h, (B) rPCIS for 24 h, and (C) mPCIS for 48 h. (*P <* *0.05, **P <* *0.01 vs. 0 h. *n* = 3–6, data are expressed as mean ± SEM).

To determine if the early-onset of fibrosis is induced in PCIS, gene expression of various fibrosis markers was investigated. After 24 h of incubation, the gene expression of an early marker of fibrosis, *HSP47,* was elevated in hPCIS when compared with hPCIS directly after slicing. *HSP47* steadily increased up to 72 h. Furthermore, when compared with hPCIS after preparation, *SYN* gene expression significantly increased in hPCIS after 48 h and was even higher after 72 h of incubation. Conversely, *ELA* expression was decreased after 48 hr incubation (Fig.7E) and *αSMA* expression was downregulated after incubation up to 72 h compared to freshly prepared PCIS (Fig.5A). Furthermore, *COL1A1, CTGF,* and *FN2* expression was not affected during incubation of hPCIS (Fig.5A).

After 24 h of incubation of rPCIS, the gene expression of *Hsp47* and *Fn2* was significantly increased compared to PCIS directly after slicing. Similar to hPCIS*, αSma* was downregulated, whereas *Col1a1*, *Ctgf,* and *Syn* expression was unaffected after 24 h of culture (Fig.5B).

As was found in rPCIS the gene expression of *Hsp47* and *Fn2* was significantly increased in mPCIS after 24 h, which increased even further up to 48 and 72 h of incubation. *Ctgf* expression was only increased after 72 h of incubation in mPCIS. In contrast to the gene expression of *αSma* and *Col1a1,* which was significantly downregulated up to 72 h in mPCIS (Fig.5C). *Syn* expression remained unchanged during incubation in both rPCIS and mPCIS.

### Addition of TGF-β1

**Figure 6:**
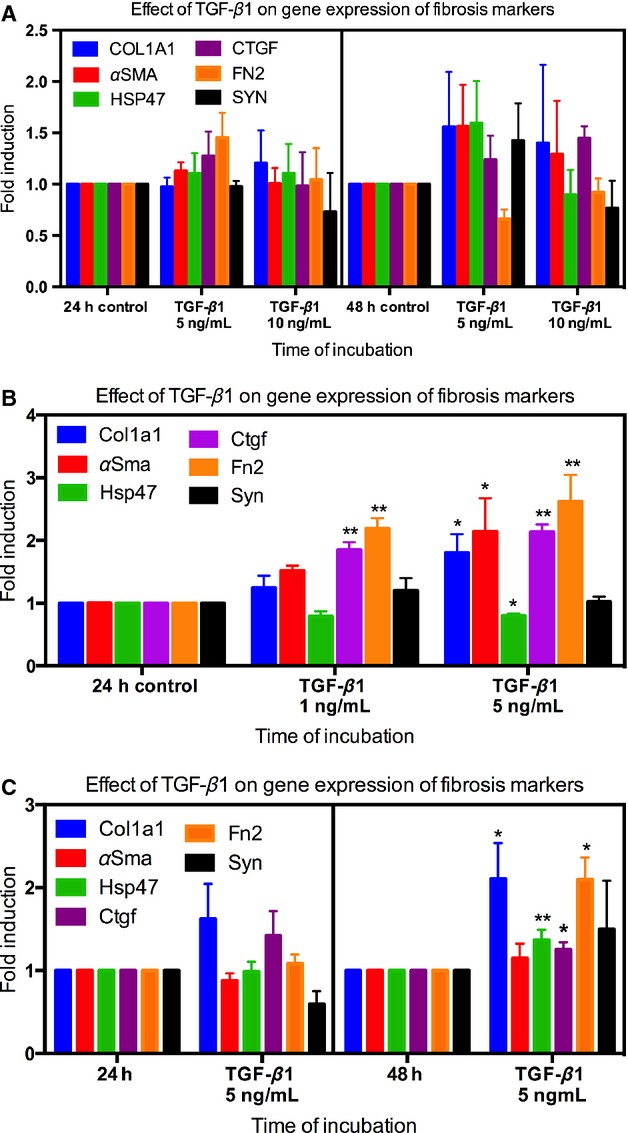
Gene expression of fibrosis markers in hPCIS, rPCIS, and mPCIS after long-term incubation with TGF-*β*1. The gene expression of fibrosis markers *COL1A1*, *HSP47*, *αSMA*, *CTGF*, *FN2,* and *SYN* after incubation of (A) hPCIS for 48 h with 5 ng/mL and 10 ng/mL TGF-*β*1, (B) rPCIS for 24 h with 1 ng/mL and 5 ng/mL TGF-*β*1, and (C) mPCIS for 48 h with 5 ng/mL TGF-*β*1. (*P <* *0.05, **P <* *0.01 vs. control. *n* = 3–5, data are expressed as mean ± SEM).

To study if the main fibrogenic factor TGF-*β*1 was able to induce fibrogenesis in these models, PCIS were incubated with TGF-*β*1. The gene expression of none of the investigated fibrosis markers was affected in hPCIS when incubated for 48 h with up to 10 ng/mL TGF-*β*1 (Figs.6A and7E). However, in rPCIS, when incubated for 24 h with 1 ng/mL TGF-*β*1, *Ctgf,* and *Fn2* were significantly upregulated compared to control and remain elevated in the presence of 5 ng/mL TGF-*β*1. Meanwhile, *αSma and Col1a1* expression were significantly increased compared to the 24 h control only, when incubated with 5 ng/mL TGF-*β*1. Interestingly, *Hsp47* expression in rPCIS tended to decrease with 1 ng/mL TGF-*β*1 and was significantly downregulated when adding 5 ng/mL TGF-*β*1 during culture (Fig.6B). In mPCIS after 48 h of incubation in the presence of 5 ng/mL TGF-*β*1 the gene expression of *Col1a1*, *Fn2, Hsp47,* and *Ctgf* was significantly upregulated compared to the 48 h control (Fig.6C). However, *Syn* expression was not affected in both rPCIS and mPCIS in the presence of TGF-*β*1.

**Figure 7:**
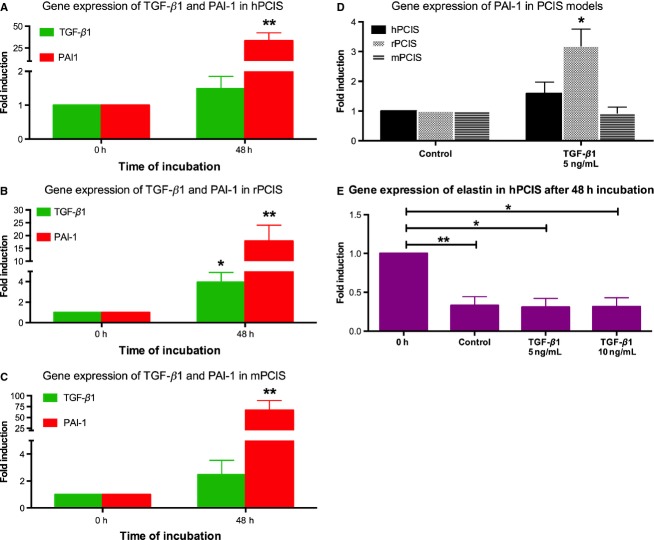
Gene expression of *TGF-β1* and *PAI-1* in hPCIS, rPCIS and mPCIS after long-term incubation with TGF-*β*1. The gene expression of fibrosis markers *TGF-β1* and *PAI-1* after incubation of (A) hPCIS for 48 h, (B) rPCIS for 24 h, and (C) mPCIS for 48 h. (D) The gene expression of *PAI-1* in PCIS models after incubation (48 hr with hPCIS and mPCIS, 24 hr with rPCIS) with 5 ng/mL TGF-*β*1. (E) The gene expression of *ELA* in hPCIS model after incubation for 48 hr with and without 5 ng/mL and 10 ng/mL TGF-*β*1 (*P <* *0.05, **P <* *0.01 vs. 0 h or control. *n* = 3–5, data are expressed as mean ± SEM.

Both *TGF-β1* and *PAI-1*, the specific downstream marker of TGF-*β*1 signaling (Kuang et al. 2007), gene expression were investigated. Only in rPCIS the *Tgf-β1* was significantly increased (Fig.7B). However, the gene expression of *PAI-1* was increased dramatically in all species suggesting activation of the TGF-*β*1 pathway (Fig.7A–C). When slices were incubated in the presence of TGF-*β*1, the gene expression *PAI-1* was not further increased, except for rPCIS (Fig.7D).

## Discussion

Intestinal fibrosis is a complicated condition, caused by extensive chronic inflammation or injury of the bowel. Although there are some animal models for IF, most of them are limited in their relevance to human disease and have some definite disadvantages, such as animal discomfort and long time needed to establish the fibrosis state (Rieder et al. 2012). Until now, there are no antifibrotic drugs available and relevant models are needed. As reviewed before, precision-cut tissue slices can provide a good model to study the early-onset of organ fibrosis (Westra et al. 2013), and can also considerably reduce the number of animals used in intestinal fibrosis research. The aim of this study was to develop a method for studying the early-onset of IF by using PCIS from various species, namely human, rat, and mouse.

### Viability by ATP content of PCIS

Precision-cut intestinal slices have been used previously to study drug metabolism and toxicity in the intestinal tract. In these studies, hPCIS were used to investigate the regulation, expression and capacity of metabolic enzymes, transporters and receptors indicating that PCIS can mimic the intestine in vivo (Van De Kerkhof et al. 2006; Van de Kerkhof et al. 2008; Khan A.A. et al. 2009; Khan et al. 2010). Previously, hPCIS were incubated for a relatively short period of time (up to 24 h). To study the onset of fibrosis, we incubated PCIS for a longer period (rPCIS up to 24 h, mPCIS and hPCIS up to 72 h). ATP is used in precision-cut tissue slices from different organs as general marker of viability. To establish this in PCIS the morphology during incubation was compared to the ATP content in the slices. Morphological scoring (according to the modified Park score (Park et al. 1990; Roskott et al. 2010)) of mPCIS, rPCIS, and hPCIS showed that the ATP is related to the morphological integrity of the tissue. Therefore, ATP levels in PCIS are used as a general marker for morphological integrity in different species in this study. In addition, the sequence of losing cell integrity in PCIS from different species is similar, indicating that the same processes take place in the PCIS from all studied species. Epithelial and stromal cell damage was observed first, indicating that these cells are the most vulnerable to ischemia. This is also shown in the original article describing the Park score (Park et al. 1990). However, more specific markers for the different cell types in the PCIS are necessary to obtain information on the viability of these cells in PCIS during culture.

The decline of ATP content found in our study was also observed in other studies with rPCIS (Van de Kerkhof et al. 2007) and hPCIS (De Graaf et al. 2010). Moreover, PCIS from the different species behave differently during culture. The ATP content in PCIS directly after slicing was comparable in all species. Striking is, however, the species difference of the maximal incubation period of the PCIS. In rPCIS after 24 h incubation ATP content was less than 50% compared to directly after slicing, in contrast to mPCIS and hPCIS, where this value was reached only after 72 h. One of the factors may be the production of reactive oxygen species (ROS). During slicing and storage before the start of the incubation, the PCIS are subjected to ischemia. Upon culturing, the PCIS are reoxygenated. It is known from studies in biopsies that in the rat intestine xanthine oxidase (XO) steeply increases during ischemia, which is in contrast to the human intestine (Bianciardi et al. 2004). Upon reoxygenation, XO will lead to ROS and subsequently to cell and tissue damage (Bianciardi et al. 2004). Furthermore, *Tgf-β1* gene expression was also increased in rPCIS, possibly subsequently also TGF-*β*1 protein and this could lead to the production of ROS (Rhyu et al. 2005; Yan et al. 2014). This might explain why rPCIS deteriorate faster in culture than mPCIS and hPCIS. Future studies will be performed to determine ROS production in PCIS of different species.

### Gene expression of fibrosis markers

The aim of our study was to induce the early-onset of fibrogenesis, which could be triggered by the loss of cell integrity over time. Therefore, we studied the gene expression of different fibrosis markers, often linked to the protein expression (Westra et al. 2014a,b). In addition, TGF-*β*1 was added to the PCIS to establish if one of the main inducers of fibrogenesis is also effective in PCIS.

In hPCIS cultured up to 72 h, *HSP47* gene expression was elevated. Other studies have identified *HSP47* as a potential early marker of IF (Taguchi and Razzaque 2007; Honzawa et al. 2010). It has been demonstrated that the serum level of *HSP47* is higher in CD patients, who are prone for IF, compared to those with ulcerative colitis, an intestinal disease that rarely leads to IF, and to control patients (Taguchi et al. 2011). Moreover, Honzawa et al. showed that *HSP47* expression contributes to IF in CD (Honzawa et al. 2014) and in the IL-10^−^/^−^ mouse model of IF, HSP47 plays an essential role (Kitamura et al. 2011; Rieder et al. 2012). Therefore, *HSP47* is a biomarker for IF and furthermore used ex vivo in hPCIS to study the efficacy of antifibrotic drugs(Taguchi et al. 2011).

*SYN,* a marker of stellate cells (Cassiman et al. 1999), was also upregulated in hPCIS. As was reported before by Rieder et al. (Rieder and Fiocchi 2008) stellate cells are present in the intestine and may contribute to the fibrotic process. As was shown before with hepatic stellate cells (HSCs), if the liver is injured, HSCs change their phenotype to an activated state, start to express among others *αSMA*, and to synthesize proinflammatory cytokines and ECM proteins (Li and Friedman 1999). Intestinal stellate cells seem to display the same basic morphological, phenotypic, and functional characteristics as the hepatic stellate cell and recently have been isolated and cultured from mesenteric fibrotic tissue from a patient with a fibrotic carcinoid tumor (Kidd et al. 2007; Fiocchi and Lund 2011). Therefore, the increase in stellate cell number and *HSP47* induction up to 72 h in hPCIS during culturing indicates that slices can be used as a tool to study the early stage of fibrosis.

The decrease in *αSMA* expression up to 72 h in hPCIS might be explained by a loss of fibroblasts, which was also found in other organ slices (Westra et al. 2013). In hPCIS, none of the other fibrosis markers (*COL1A1, FN2, and ELA*) were increased during culturing, even with the addition of increasing concentrations of TGF-*β*1. This may indicate that the TGF-*β* pathway cannot be induced, or is already activated due to the operation and procurement of the tissue. Moreover, it should be noted that in the intestine, the resident macrophages are mainly in the muscularis (Smith et al. 2011), which was removed from the human intestinal tissue before hPCIS were prepared. Although the mechanism of IF in human is still unknown, the spontaneous early fibrosis may require macrophages to develop. Future studies with specific inhibitors of the TGF-*β* pathway will elucidate if the pathway is already activated. Furthermore, maybe an additional trigger such as PDGF or the presence of the inflammatory cytokine TNF*α* is necessary to induce fibrosis in hPCIS, which has been reported in liver fibrosis and idiopathic pulmonary fibrosis (Kropski et al. 2012; Basaranoglu et al. 2013). In future experiments with PCIS it will be elucidate if a second hit is necessary. In human liver slices, not only TGF-*β*1 but both potent fibrogenic factors PDGF and TGF-*β*1 are necessary to induce gene expression of fibrosis markers (Westra et al. 2011). Research is currently ongoing to further induce the gene expression of fibrosis markers in hPCIS.

We have investigated the gene expression of *ELA* in the hPCIS. Although it has been reported that *ELA* was activated by TGF-*β*1 in lung fibroblasts (Kuang et al. 2007), there is no reports of the involvement of elastin in fibrosis in other organs. In hPCIS, *ELA* was not induced during incubation with or without TGF-*β*1. This may indicate that the activation of elastin is lung specific.

In rPCIS, both *Hsp47* and *Fn2* were increased after 24 h of culture. These results imply that the early-onset of fibrogenesis is indeed induced in rPCIS during culture up to 24 h. Addition of TGF-*β*1 further increased *Fn2* expression, but not *Hsp47* that was downregulated by TGF-*β*1. This may be explained by the fact that for the early fibrosis marker *Hsp47*, the maximum gene expression was reached earlier by adding TGF-*β*1 during rPCIS incubation than in the control incubation of rPCIS. This is in accordance with the results of *Col1a1*, the marker of fibrosis, that was only elevated in rPCIS by TGF-*β*1, as was also seen in other organ slices (Westra et al. 2011). Similarly, *Ctgf* expression was only elevated in rPCIS after the addition of TGF-*β*1, this is in line with the function of Ctgf in the TGF-*β* pathway (Brigstock 2010). As was assessed in hPCIS, *αSma* expression was also decreased in rPCIS, indicating again that fibroblasts may be lost during culture. However, *αSma* expression was increased in rPCIS by TGF-*β*1. This may suggest that (myo)fibroblasts are activated by TGF-*β*1 in rPCIS.

In accordance with the results in rPCIS, prolonged culture of mPCIS induced *Hsp47* and *Fn2* expression, but *αSma* was decreased during culture up to 72 h. However, unlike rPCIS and hPCIS, where *Col1a1* was unchanged during culture, in mPCIS the gene expression of *Col1a1* was significantly decreased. In an ex vivo model of liver fibrosis (Westra et al. 2014b), *Col1a1* was also downregulated after 24 h incubation, but increased after 48 h incubation due to activation of the wound repair system (Vickers and Fisher 2005). In mPCIS, probably longer incubation in the presence of profibrotic cytokines is needed to initiate the wound healing process and fibrosis. Interestingly, only in mPCIS, *Ctgf* gene expression was upregulated after 72 h incubation, this is an indication that the TGF-*β* pathway was activated after long-term incubation. Furthermore, addition of TGF-*β*1 to mPCIS-induced gene expression of *Col1a1*, *Fn2*, *Ctgf,* and even *Hsp47* that was downregulated in rPCIS. *αSma* expression was not affected by TGF-*β*1 in mPCIS. In contrast to hPCIS, *Syn* was not induced in rPCIS and mPCIS, not even in the presence of TGF-*β*1. This suggests differences between species in the proliferation of intestinal stellate cells.

All these results imply that in rPCIS and mPCIS there was a spontaneous induction of early fibrogenesis, which can be measured by gene expression of *Hsp47* and *Fn2*. Our result of the early-onset of fibrosis in PCIS are in line with the results in a liver fibrosis model using precision-cut liver slices, which we already successfully used in studying antifibrotic compounds (Westra et al. 2014b). Future studies with specific signaling pathway inhibitors will be performed to elucidate why during culturing of PCIS spontaneous induction of these markers occurs. TGF-*β*1 was able to even further stimulate the onset of fibrosis in rat and mouse, indicating the importance of TGF-*β*1 as profibrotic stimulus in rodents. Upcoming experiments have to clarify if the TGF-*β*1 pathway is involved in the spontaneous activation of early fibrogenesis in PCIS. Moreover, we found clear species differences in the early-onset of fibrosis, therefore, we are currently performing studies, among others by staining different intestinal cell types, to elucidate these difference.

## Conclusion

We successfully developed a relevant ex vivo intestinal model in both rodent and man to study the early-onset of intestinal fibrosis. In rat and mouse PCIS, TGF-*β*1 was able to even further increase the gene expression of fibrosis markers. The gene expression of *HSP47* in human and rodent PCIS, and in rodent PCIS also *Fn2*, can be used as early markers of fibrosis. These results open the opportunity to test the efficacy of antifibrotic drugs in both human and rodents in an ex vivo physiological model. The model also provides the opportunity to study the fibrogenesis in different regions of the intestine. Furthermore, the mechanism of fibrosis in an ex vivo model of early fibrogenesis in different species can be determined. The research is currently expanding to the diseased human (fibrotic) intestine.

## Conflict of Interest

None of the authors has anything to declare.
